# Assessment of dental students’ communication skills with patients

**Published:** 2016-01

**Authors:** MAHTAB MEMARPOUR, LEILA BAZRAFKAN, ZAHRA ZAREI

**Affiliations:** 1 Prevention of Oral and Dental Disease Research Center, School of Dentistry, Shiraz University of Medical Sciences, Shiraz, Iran;; 2 Quality Improvement in Clinical Education Research Center, Education Development Center, Shiraz University of Medical Sciences, Shiraz, Iran;; 3 Student Research Committee, School of Dentistry, School of Dentistry, Shiraz University of Medical Sciences, Shiraz, Iran

**Keywords:** Communication skill, Relationship, Dental Student

## Abstract

**Introduction:**

Establishment of effective communication between the clinician and patient is essential in order to increase the effectiveness of treatment. These skills have been less investigated among dental students. This study aimed to evaluate communication skills of dental students in Shiraz with patients through direct observation, patients' perspectives and students' self-assessments.

**Methods:**

This cross-sectional study enrolled the fifth and sixth year dental students and one of each student’s patients who was chosen using simple random sampling method. We used a checklist for data collection. Students’ communication skills were assessed at three steps of the student-patient interview – at the beginning of the interview, during the interview, and at the end of the interview. The checklist was completed by three groups: 1) an observer, 2) the patient and 3) the student, as self-assessment. The validity of the checklist was confirmed by clinical professors and the reliability was determined by Cronbach's alpha test. Data were analyzed using descriptive statistics and Student’s t test. A repeated measure MANOVA was used to compare the mean communication skills in the researcher, patients, and students at each step of the patient interviews.

**Results:**

There were 110 students (mean age: 22.3±8.4 years) and 110 patients (mean age: 32±8.8 years) who completed the checklists. Overall, the communication skills of dental students were rated as good according to the patients. However, the observer and student participants rated the skills at the moderate level. We observed significant differences between communication skills in all three groups and in the three steps of the patient interviews (p<0.001). According to patients' beliefs and students' self assessments, there were no differences between male and female students in communication skills in the three steps of the patient interviews (all p>0.05). However from the observer’s viewpoint, female students showed better communication skills during the interviews (p=0.001).

**Conclusion:**

There was a degree of failure in communication skills of dental students with patients in the interview process. It will be necessary that communication skills be taught, particularly for students involved in clinical practice.

## Introduction

Teaching communication skills as a part of the dentistry curriculum leads to an increase in the dentist's ability to understand the patient's needs, comments and responses to these needs (1). The nature of the dental treatment is often associated with the patients’ stress. Effective communication with patients reduces the patients’ anxiety, and increases the patients’ interest to accept dental treatments and perform the dentists’ recommendations (2, 3).

For many years, the communication model of the relationship between physician and patient has focused on the physician's role. However, the patient's role and needs are important. Dentists encourage active participation by patients during the interview process to enable patients to express any emotional or psychological needs. Changing the physician-based model to a patient-based model plays an important role in increasing the patient’s satisfaction with treatment (4, 5).

Several studies have evaluated the importance of communication skills training of the health care members (6-9)and the effectiveness of teaching these skills to dental students(10-13). Van Der Molenet et al. evaluated the efficacy of a communication skills training program to manage the patients’ stress and fear of dental treatments. They reported that communication skills training effectively increased the dental students’ knowledge and behavior. This training promoted the students' awareness about their limitations and capacity to communicate with patients (3).

A regular program on communication skills training affects the acquisition of the skills. Cannick et al. have shown that short term intervention in skills was neither successful, nor effective (10). The level of education also influences the communication skills. Most medical students use the physician based role model during the last year of their studies (11). Kitzman interviewed 50 physicians who were previously diagnosed with serious illnesses. In this study, it was reported that physicians recognized the importance of communication skills after they were ill and referred as patients (12).

Several studies have used different methods to evaluate communication skills among students (10, 13, 14-15). The assessment by direct observation of communication skills training, has been used in several studies (16-19). However, since this method is only one aspect of assessment, in addition to direct observation, studies have used other methods such as self assessment and patient assessments as well (20, 21).

With regard to the importance of assessment of communication skills training and lack of formal education and assessment of these skills, this study aimed to evaluate communication skills of Shiraz dental students with patients using the researcher’s direct observation, patients' perspectives and students' self-assessments. 

## Methods

This research protocol was approved by the Human Ethics Review Committee of the Faculty of Dentistry, Shiraz University of Medical Sciences. Participants included all undergraduate dental students enrolled in the last two years of their studies (fifth and sixth year students) in dental school, Shiraz, Iran. For this cross-sectional study (2011-2012), we enrolled 110 dental students and for each dentist, one of their adult patients (n=110) was selected using simple random sampling method. 

Data were collected using a checklist that consisted of 22 questions derived from the Calgary-Cambridge guidelines (22). Questions were asked about the dentist's beginning of the interview with the patient, performance during the interview and termination of the interview. The responses were evaluated by a three-point Likert scale that ranged from good (score of 1) to moderate (score of 2) and poor (score of 3). The checklist was used for all the three groups. There were two additional questions for the student's checklist pertaining to the necessity of adding communication skills to the educational curriculum.

The validity of the checklist was confirmed by clinical professors. The reliability coefficient according to Cronbach's alpha was as follows: r=0.82 for the researcher, r=0.85 for patients, and r=0.92 for students.

Three groups participated in the study and completed the check list: the observer 1) researcher, 2) the patients and 3) dental students. The observer (researcher) attended daily meetings from 9 am to 12 pm as a student assistant or observer (with the permission of the head of the department). The researcher subtly observed the students during the patient interviews and completed the dentist-patient checklist from the point of view of the observer. Students who communicated adequately received good scores. Those who received 50% and 10% favorable scores for communication skills were classified as moderate and poor communicators, respectively. 

The patients were chosen from different departments in the dental school. Patients of pediatric, orthodontic and oral radiology departments were excluded since the pediatrics` and orthodontics patients were too young to participate in the study. Moreover, the radiology patients’ interaction with the students was less than 5 minutes; therefore, the students didn’t have enough time to communicate with them. At the end of the patient's dental treatment and at the time the patient left the ward, the study aim was explained to each patient who participated. All of them provided their informed consents in writing. After expressing their consent, the checklist was subsequently completed by the patient as an evaluation of the dental student's communication skills. 

Dental students completed the checklist. This checklist was completed by the student who performed the dental treatment in order to know his or her view regarding the use of communication skills in the patient interviews. In order to prevent bias in the results, at the beginning of the study the students were unaware of the process. However, the students were informed after the questions were answered. Students were assured that completion of the forms did not influence their course assessment and all information was confidential. In addition, the completed checklists gave the students an idea of their performance during the treatment. Informed consents were signed by the students to enable them to use the checklists for data gathering.

To prevent inaccuracy in completing the checklists, the researcher was present to answer any questions raised by respondents (patients and students) while they were completing the forms.

All data were analyzed using SPSS, version 14 (SPSS Inc., Chicago, IL.). Data were presented using mean±SD or frequency (%) as appropriate. We compared the mean communication skills between males and females using student’s t-test. A repeated measure MANOVA was used to compare the mean communication skills rated by researchers, patients, and students at each step of the patient’s interview. 

## Results

In total, 110 students consisting of 47 (42.7%) males and 63 (57.3%) females participated in the study. The mean age of the students was 22.3±8.4 years. One patient was enrolled for each student with the intent to evaluate the student communication skills. A total of 110 patients participated in the study, including 30 (27.3%) males and 80 (72.7%) females with a mean age of 32±8.8 years. In terms of education, the majority of the patients’ education [n=99 (90%)] were less than a diploma and 11 (10%) had a diploma. Table 1 shows the frequency of answers to the checklist according to the observer, patient and student beliefs.

**Table 1:** The mean percentage of score reported by the observer, patients and students for the three interview steps with patients

**Communication skills score**	**Three interview steps**			
		**Beginning of interview**	**During the interview**	**End of the interview**
**Good**	Observer	20.9%	24.6%	27.7%
	Patient	59.02%	76.7%	73.3%
	Student	53.4%	47%	34%
**Moderate**	Observer	47.7%	53.2%	62.1%
	Patient	9.8%	14.3%	19.4%
	Student	32.5%	52.6%	55.1%
**Weak**	Observer	31.4%	20.1%	10.2%
	Patient	31.2%	9.1%	8.9%
	Student	14.1%	4.3%	10.6%

The main questions of checklist at three steps of the student-patient interview included: At the beginning of interview: greeting, asking the patient's name, introducing themselves to patient, attention for patient comfort, explaining the aim of the interview. During the interview: explaining the treatment with easy to understand words, asking questions, encouraging the patient to speak, friendly communication, support and sympathy with the patient, response to patient questions, respect for patient's beliefs, conducting interviews, active listening to patients. At the end of the interview: answering additional questions, assess patient's understanding of the interview, summation results of the interviews, announce end of interview.

Regardless of the steps of the interview process, there was a significant difference between observer, patients and students in terms of overall mean communication skill; (p<0.001; Table 2). The highest score was given by patients (56.44±6.07), followed by students (51.57±5.94) and the observer (46.68±7.53).

**Table 2:** Mean±SD of dental students in communication skills according to the observer's, patients' and students' scores (*Statistically significant p<0.05)

**Group**	**Mean** **±** **SD**	** p **
**Observer**	48.63±7.53	0.001*
**Patients**	56.44±6.07	
**Students**	51.57±5.94	

A comparison between the mean scores for the observer, patients and students in each step of the interview showed a significant difference between the groups (p<0.001; Table 3). The beginning of the interview had the highest score given by the patients (11.90±1.68) followed by students (9.51±1.21) and the observer (7.56±1.63). Mean scores for the performance of the interview were as follows: patients (35.12±3.84), students (31.13±4.01) and observer (28.25±4.46). The end of the interview mean scores were: patient (12.20±2.03), observer (11.04±1.89) and students (10.84±1.98).Overall, the communication skills of dental students was rated as good according to the patients; however, the observer and students rated this skill as moderate level. According to the scores given in the three steps of the interview process, the communication skill of students was assessed as moderate (63%).

From the patients and students’ point of view, there was no significant difference between male and female students in communication skills for any of the three steps. There was no significant difference between male and female students at the beginning and end of the interview from the observer's point of view. However, the observer gave a higher score to female students (29.42±4.95) than males (26.68±3.13; p=0.001) during the interview. Also the fifth grade students received higher scores than sixth grade students; (p=0.001(. A total of 65% of dental students believed that training on communication skills was necessary during their education, particularly for clinical practice. However, 31% of them believed in the importance of skill training to be moderate and 4% stated that training on communication skills was not required.

**Table 3:** Mean± SD of dental students' communication skills according to observer's, patients' and students' scores in the three interview steps (*Statistically significant p<0.05)

**Communication skills step**	**Group **			**p**
	**Observer **	**Patients (n=110)**	**Students (n=110)**	
**Beginning of interview**	7.58±1.63^A^	11.90±1.68^B^	9.57±1.21^C^	0.001*
**During interview**	28.25±4.46^ A^	35.12±3.84^ B^	31.13±4.01^ C^	0.001*
**End of interview**	11.04±1.89^ A^	12.20±2.03^ B^	10.84±1.98^ C^	0.001*

*Data were presented as Mean ±SD and were analyzed via ANOVA

In each row of table, mean values with different capital letters in superscript are statistically different (using LSD post hoctest).

## Discussion

This study showed significant differences between beliefs of the observer, patients and students in assessing communication skills, which indicated poor validity for each method if used alone for the evaluation of communication skills. The students and observer gave almost similar scores while the patients expressed satisfaction with the dental students' communication skills during the interview. In agreement with our result, previous studies revealed that most patients were satisfied with their communication with the physician (17, 23). One study reported that patients believed that the physician spent an adequate amount of time during the interview; however, the observer’s opinion was in contrast with them (17). In agreement with these studies, we found that most patients were satisfied with the students during the interview process and believed the students had a friendly relationship with them. The observer reported that most of the students performed weakly in this area. This weakness was also observed in the interview that pertained to asking questions or active listening. This might be related to the fact that patients were unfamiliar with their rights (24). Additionally, culture, socioeconomic class and level of education influenced the patients' judgments. Differences between students and the observer's ideas might affect the results.

The current study showed that dental students' communication skills were in the moderate level; this is in the same line with Molaei et al.’s findings (9). However, one study scored this skill as weak, which might be related to the lack of communication skills training by students (6). Other factors such as the stressful condition of the treatment procedure, symptoms of burnout or exhaustion of students, emotional fatigue, lack of leisure time, exam pressure and the large number of books and learning activities might influence the students' communication skills (24-26).

In the present study, most patients believed that students conducted the interviews respectfully with regards to the patients' ideas and did not be little them. This result was confirmed by the observer. This might be attributed to the patient participation in the interview process. Acceptance of the patients’ ideas does not mean agreement with all the patients’ beliefs. The interview process could be performed with respect to the patients' rights and correct misunderstandings by stating the clinicians' perceptions (22, 25).

The observer reported that more than more than a half percent of the students did not encourage patients to talk and occasionally interrupted the patients while they were speaking. This might be the result of using traditional methods to obtain the patient histories and the students’ negligence to show concern for patients’ ideas. In agreement with these results, a number of previous studies reported that students had moderate or poor skills in encouraging the patients to talk (26-28). The clinician might use a similar method to interview all patients with different problems. This might have been related to the use of a physician-based model for interviewing in which patients are not allowed to express their problems which leads to an inaccurate interview process (28).

Student introductions to their patients received the weakest score by both the observer and the students. As in the present study, Rahman et al. reported that the weakest point of the students was at the beginning of the interview, while explaining the aim of the interview and introducing themselves to the patients (29).

Our findings showed that the observer, patients and students confirmed that students used simple, easy words and explained the procedure in an understandable way for the patients. This might be attributed to the emphasis placed by professors on not using professional words for patients. However, in a study by Marteau et al. students used complicated medical words during patients' interviews (30).

The current study showed that both female and fifth grade students conducted better interviews with the patients than males and last year students, which agreed with a previous study (9). Haidet et al. showed that with increase in the level of education, the attitude of medical students to use communication skills decreased (11). The current study also showed a significant difference between the mean communication skills at the beginning, the performance and at the termination of the interview with patients. This might be related to the lack of education of students in this skill. Thus the majority of students believed that training on communication skills is necessary during their education, particularly for clinical practice. Hottel and Hardigan as well as Hannah et al. have shown that communication skills are an acquired skill and should a part of the course content in this field (1, 31, 32).

A potential limitation of this study was the level of boredom and lack of sufficient accuracy by students and patients in completing the checklist. To overcome this problem, the researcher explained the aim of the study and was present during completion of the checklists. Also, the selection of patients from different wards who had undergone a variety of treatments might have influenced the results. Patients’ satisfaction regarding the students’ communication skills might be influenced by their level of education. Therefore, a future study should consider enrolling patients from different socioeconomic classes and education levels.

## Conclusion

Dental students had a moderate level of communication skills with patients. We observed a significant difference between the mean of students’ skills scores given by the observer, patients and students. Patients expressed a more positive view regarding the students’ skills compared to the observer and students. With regards to the importance of communication skills, these skills should be considered when developing the curriculum content and assessed as a part of evaluation in clinical skills. 
